# Recent Developments to the SimSphere Land Surface Modelling Tool for the Study of Land–Atmosphere Interactions

**DOI:** 10.3390/s24103024

**Published:** 2024-05-10

**Authors:** George P. Petropoulos, Christina Lekka

**Affiliations:** Department of Geography, Harokopio University of Athens, El. Venizelou 70, 17671 Athens, Greece; clekka@hua.gr

**Keywords:** land surface interactions, SVAT, SimSphere, triangle, geoinformation

## Abstract

Soil–Vegetation–Atmosphere Transfer (SVAT) models are a promising avenue towards gaining a better insight into land surface interactions and Earth’s system dynamics. One such model developed for the academic and research community is the SimSphere SVAT model, a popular software toolkit employed for simulating interactions among the layers of vegetation, soil, and atmosphere on the land surface. The aim of the present review is two-fold: (1) to deliver a critical assessment of the model’s usage by the scientific and wider community over the last 15 years, and (2) to provide information on current software developments implemented in the model. From the review conducted herein, it is clearly evident that from the models’ inception to current day, SimSphere has received notable interest worldwide, and the dissemination of the model has continuously grown over the years. SimSphere has been used so far in several applications to study land surface interactions. The validation of the model performed worldwide has shown that it is able to produce realistic estimates of land surface parameters that have been validated, whereas detailed sensitivity analysis experiments conducted with the model have further confirmed its structure and architectural coherence. Furthermore, the recent inclusion of novel functionalities in the model, as outlined in the present review, has clearly resulted in improving its capabilities and in opening up new opportunities for its use by the wider community. SimSphere developments are also ongoing in different aspects, and its use as a toolkit towards advancing our understanding of land surface interactions from both educational and research points of view is anticipated to grow in the coming years.

## 1. Introduction

The land and atmosphere interact in complex ways through many biophysical and biogeochemical feedbacks, operating at various spatial and temporal scales [1]. The land surface interacts with the atmosphere through the exchange of momentum, water, and carbon dioxide. Thus, land surface interactions (LSIs) can be summarised by the energy balance, water, and carbon cycles and the associated critical processes. As a result, LSIs influence microclimate, crop productivity, and extreme weather events such as heat waves, droughts, and floods. These interactions are important drivers of the Earth’s system, regulating the critical exchanges of mass and energy between the atmosphere and the terrestrial biosphere [2,3,4,5]. Developing knowledge on Earth’s natural processes and component interactions by recognizing its vital aspects is a key research priority, especially in the face of ongoing climate change challenges. Moreover, understanding LSIs dynamics can support the development of sustainable management strategies and policies [6]. 

Energy fluxes are typically obtained from ground monitoring networks of in situ flux measurements, which are labour-intensive and high-cost and provide limited coverage. Ground-based instruments, such as eddy covariance, provide direct measurements but face challenges for large-scale use due to a lack of spatial and temporal variability in observation coverage, instrumentation complexity, difficulties in setting up, and cost. Ground operational networks such as FLUXNET nowadays provide ground observations at no cost that support local-scale monitoring and model benchmarking, albeit with geographical inhomogeneity. In FLUXNET, the eddy covariance method is employed across all sites to quantify the carbon, water, and energy fluxes between the biosphere and the atmosphere [7,8,9,10].

Mathematical models have demonstrated their effectiveness for studying and better understanding Earth’s physical processes such as those of the energy balance, carbon, and hydrological cycles and evaluating how these processes are impacted under different climate change scenarios [11,12,13]. Various such models have been developed to study LSIs at different geographical scales. One of the most promising of these includes land–biosphere models (LBMs) [14]. These simulate physical processes on the land surface related to radiation absorption and partitioning, water and carbon cycles, and atmospheric interactions. These models are able to estimate carbon and energy fluxes, e.g., sensible heat fluxes (H), latent heat fluxes (LE), solar radiation, surface runoff, and soil moisture availability [15]. Yet, LBMs are characterised by several disadvantages, primarily due to their inherent mathematical complexity, which is a result of the dynamic, adaptive, and infinitely complex nature of life and its interactions with the environment. This complexity poses significant challenges in accurately capturing complex ecosystem relationships, resulting in models with limited predictability. A thorough review of these models can be found, for example, in [14,16,17]. 

One type of LBM includes Soil–Vegetation–Atmosphere Transfer (SVAT) models [18]. SVATs aim to describe processes that regulate mass and energy transport in the soil–vegetation–atmosphere system (radiation, water transport, and turbulence) and to provide estimates of the variables at a detailed time step [19,20]. These mathematical models provide a comprehensive understanding of the physical processes that control the movement of matter and energy in soil, vegetation, and atmosphere [20,21,22]. SVAT schemes combined with a planetary boundary layer (PBL) model are considered to be the commonly adopted model framework at regional spatial scales [23,24]. SVATs can estimate soil and vegetation conditions over time in accordance with atmospheric processes provided to them. Accurate predictions using SVAT models are critical for modelling land–atmosphere interactions, as integral components of numerical weather prediction and assimilation models [25]. Yet, there are several limitations of SVAT models. The main challenge is linked to their parameterization, due to their large number of input parameters, which requires an excessive availability of data or field measurements. The representativeness of the spatial scale is another challenge. In addition, the computational requirements for simulations, particularly for multi-layer models, can often be both time consuming and costly, prohibiting their wider applicability [26,27].

Yet, a promising path in the use of these models includes their integration with Earth observation (EO) datasets, and for this purpose, a number of data assimilation methods have been proposed [28,29,30,31,32]. The fine temporal continuity and vertical coverage of such models, together with the horizontal coverage and spectral resolution of EO data, enable more accurate predictions, informed land-use decisions, and effective strategies for climate change mitigation [33,34]. Current efforts focus on exploiting the synergies between ground-based observations, EO technology, and SVATs to develop innovative methodologies and software tools for EO-based products to support a better understanding of hydrological and biophysical cycles. 

SimSphere is a 1D SVAT model introduced by [35,36]. The model has significantly evolved since its inception, with its most recent version developed in Java by [37]. An overview of the latest model version can be found in [19,38,39]. In brief, a number of variables are simulated by the model, including surface energy fluxes at the soil surface as well as in, around, and above the vegetation canopy, the transfer of water within the soil and the plants, CO_2_ fluxes between the atmosphere and the plants, surface O_3_ fluxes, and a variety of other parameters. SimSphere’s applicability has already been demonstrated in various ecosystems worldwide, and results confirm its promise as a tool to study LSIs. Indeed, the successful application of the model to various ecosystem types [40] confirms its ability to respond to the description of the complex physical processes of the natural environment [41,42]. Furthermore, the extensive number of sensitivity analysis (SA) experiments that have been carried out in the model confirm its architectural coherence and stability [43,44], which enhanced its architectural design [19]. In addition to its use as a stand-alone modelling tool, SimSphere has been widely adopted for its ability to be integrated collaboratively with satellite-based data, providing valuable spatial and temporal predictions of key parameters related to energy and water fluxes [44]. This integration allows for the quantification of spatially variable fluxes and provides a mechanistic framework for the extrapolation of small-scale observations to regional fluxes. It also deals with challenges related to sub-grid variability and the impact of spatial heterogeneity on carbon, water, and heat flux sources and sinks [41,45]. Various space agencies have previously explored different variants of this technique to develop operational products, as indicated by [46,47]. Since its first release, this model has undergone substantial architectural and functional development, and its application has been extensively proven in a variety of multidisciplinary scientific studies [19,37]. In addition, SimSphere has gained widespread usage as an educational toolkit to study LSIs, being used by several (at least seven) universities worldwide. 

All in all, from the above, it becomes evident that SimSphere is a well-known SVAT model, and it has been identified as a valuable tool for the study of LSIs for research and education purposes alike. Its continuous development from different facets, its successful application in a variety of ecosystems, and its ability to be used in conjunction with advanced EO data over decades provide further evidence of its recognition as a modelling tool of LSIs by the community. SimSphere’s recently refined design, coupled with its worldwide adoption, makes it a suitable model for research in exploring intricate physical processes and mitigating climate change [19]. 

Given the interest in SimSphere so far from both an educational and research point of view, providing a review on its use is very timely. Such a review not only helps to showcase in a single point of reference its use in a variety of applications and disciplines so far but also paves the way of future work linked to the model from multiple perspectives, including its architectural design and emerging applications. As such, this review also addresses key research priorities identified today by global organisations linked to the development of methodologies and modelling tools that can assist in advancing our understanding of our terrestrial biosphere and ensuring a more sustainable environment [5,48]. The adverse impacts of climate change further underscore the necessity for innovative and adaptive methodologies that will support building a sustainable future, as acknowledged, for example, at the European scale by the European Union’s environmental goals and initiatives [49]. Thus, the present SimSphere review can place SimSphere at the forefront of activities in progress globally linked to addressing global challenges. 

In this context, the present article’s aim is two-fold: (1) to provide a comprehensive critical overview of the model’s recent use by the scientific and wider user community; and (2) to provide information about recent software developments implemented in the model, which have resulted in improving its capabilities. Information on the current developments to the model and the identification of the directions in which future work should be focused on concerning its use is very timely, given the global interest in the model by the academic and scientific community via its use both as an educational and research tool alike. 

## 2. SimSphere’s Modelling Architecture

This section provides an overview of the SimSphere model’s basic architectural design. For an extensive discussion on the model’s principles, the reader may refer to [19,37,50]. The latest model version can be accessed at no cost through the Department of Meteorology at Pennsylvania State University, USA (https://courseware.e-education.psu.edu/simsphere/) (accessed on 20 January 2024). 

SimSphere is a time-dependent, two-stream model designed to simulate soil–vegetation–atmosphere interactions and calculate fluxes across a surface composed of fractional segments of bare soil and vegetation [19]. It is a one-dimensional boundary layer model operating with a plant component. The model’s scale in the horizontal domain implicitly depicts an undefined horizontal region of the Earth’s surface that is formed from a mixture of vegetation and bare soil in proportions (Fr) and (1−Fr) that range from 0 to 1.0, with Fr representing the fractional vegetation cover per unit area. As a result, the model’s horizontal scale is determined by how well its initial conditions (input parameters) correspond to the characteristics of the simulated horizontal area. SimSphere is applicable to a specific location or, at the very least, a confined area, under the condition that the characteristics of the atmosphere, surface slope, and incident radiation are evenly spread across the entire domain. 

The model is designed to operate over a full 24 h cycle, simulating the dynamic physical phenomena that occur along a vertical axis from the subterranean root zone to an elevation above the vegetation cover. The simulation is initiated at dawn (specifically at 05:30 local time) with pre-defined initial conditions to simulate the continuous dynamics between soil, vegetation, and atmosphere. Simulations are based on a number of input parameters related to time, location, vegetation, surface characteristics, hydrology, meteorology, soil properties, and the atmosphere (a total of 53 divided in 7 groups). SimSphere calculates a set of 32 variables from the initial parameters, reflecting the environmental conditions at the model site. These variables include surface energy fluxes (such as H and LE) at the soil surface and within, above, and around the canopy of the vegetation as well as carbon fluxes between the atmosphere and plants, along with the integrated surface temperature (T_s_) of the vegetation–soil mix at the scale of a grid cell [50]. 

**Figure 1:**
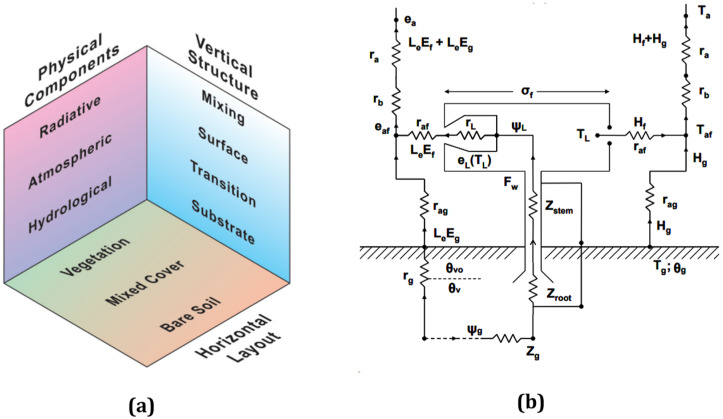
SVAT model’s different architectural components (**a**) (adopted from the SimSphere user manual (https://courseware.e-education.psu.edu/simsphere/workbook) (accessed on 20 January 2024) and the vertical structure of the plant–canopy model using an Ohm’s electrical analogy are depicted (**b**), which demonstrates how the SVAT model represents exchanges of H and LE fluxes among the atmosphere, vegetation, and bare soil. Individual model parameters which are used in Figure 1b are defined in Table 1 below (Figure 1b and Table 1 have been adopted from [36,51]).

Three primary systems can be identified within the structure of the model: the vertical, the physical, and the horizontal system (Figure 1a). The overall model structure, which includes a description of the operation of the vegetation component, is shown as Ohm’s resistance (Figure 1b) with reference to the relative parameters summarised in Table 1.

The vertical structural component corresponds to the planetary boundary layer (PBL), which is sub-divided into three layers: a surface transition layer that represents surface vegetation or bare soil; a constant flux layer at the surface; and a surface mixing layer [37]. The microclimate conditions of the model are mainly determined by the physical component, which is further divided into three groups: radiative, atmospheric, and hydrological. Another important aspect of the model is the horizontal layout, which account for spatial diversity in land (vegetation) cover. The atmospheric model layers’ depths are dynamic, which means that they vary with time. For example, high temperatures during the day will result in a significant upward transfer of heat and downward transfer of wind speed–momentum that will cause the PBL to grow to heights of 1 or 2 km, where the air from the surface is mixed. Therefore, the PBL top is defined as the top mixing layer. The constant flux represents a surface layer in which the vertical variations in heat, moisture, and wind speed fluxes do not differ by more than 10% and where certain logarithmic profile laws are thought to apply. In the model, this layer forms a series of equilibria between the mixing above and the transition layer below. Sensible heat transferred into the mixing layer causes it to warm and mix with the air above it. Latent heat conveyed into the mixing layer causes the humidity to rise within it. The transition layer is a mathematical concept specifically related to the vertical transfer of moisture and heat across the bare soil component. In the transition layer, the vertical exchange is dominated by molecular and radiative effects and wind shifts. When it comes to vegetation, an actual layer of vegetation replaces the transition layer. The temperature, moisture, and wind profiles in the bare soil or vegetation transition layers, like the surface layer, are controlled by laws that represent equilibrium between the layers below and above it. During the day, the top of the mixing layer rises in response to the H fluxes at the surface, as evidenced by a temperature inversion that traps the air in convective interaction with the surface layer. 

The substrate layer represents the depth of soil through which water and heat are transferred. The user defines water content in the soil for the root zone layer and the top layer by allocating a fraction of the field’s capacity. The model’s PBL is represented by the fractional vegetation cover (Fr) and the bare soil fraction. All of these systems are treated separately, and the resulting flows are combined at the base of the surface layer, which is typically fifty meters above ground. Through vertical heat, energy, water vapour, and CO_2_ transfer, the PBL receives almost all of its water vapour and most of its heat from the surface. These transfers are mainly driven by mechanical wind turbulence between the surface and atmosphere, and turbulent eddies caused by surface heating. The proper balance of energy fluxes at the surface of the Earth and within the plant canopy poses a challenging constraint for the model. The model begins by calculating solar radiation using a one-dimensional boundary layer. Surface albedo, solar geometry, and atmospheric transmission coefficients are used to calculate the total amount of downwelling irradiance absorbed by the substrate layer at a given date, time, and latitude and longitude location. The radiation flux calculations are designed to work under the assumption that no clouds are present, although adjustments for a specific cloud coverage ratio can be added during initialisation if necessary [50]. 

In its parameterisation, the model includes a vegetation layer between the surface of the atmosphere and the ground, when the vegetation is activated. Leaf area index (LAI) and Fr are used to quantify vegetation density. Each system functions autonomously at the canopy level when integrating representations of bare ground and vegetation to accommodate partial vegetation cover conditions. Nonetheless, there is a mutual exchange of momentum, heat, and water vapor occurring between the shared substrate beneath and the common surface and mixed layers above the canopy. For both the bare soil and vegetation, the calculation of incoming shortwave radiation, downward longwave radiation, and radiation partitioning remains consistent. Similarly, LE, H, upward longwave radiation flux above the canopy, and substrate heat flux (G) are derived as weighted averages of the vegetation and bare soil components within the SVAT model, with the vegetation fraction as the basis for weighting. In both the vegetation and bare soil portions, temperature, specific humidity at the top of the surface layer (T_a_; q_a_), and soil water content are identical. The canopy surface temperature (T_s_) is then calculated using a weighted average of upward long-wave radiation fluxes from both the vegetation and bare soil components. 

The model’s parameterisation that concerns, in particular, the plant’s stomatal resistance is a critical aspect of the modelling parametrisation process. Stomatal resistance, which represents the resistance of the vegetation to transpiration, is important in controlling the energy partitioning between the H and LE fluxes. SimSphere allows the user to choose between two options for modelling stomatal resistance: (1) the [52] parameterisation, and (2) the [53] formulation. The former has the advantage of being able to represent the broad aspects of the behaviour of stomata under the influence of sunlight and water in the soil. However, the main disadvantage is that plant hydraulics are not taken into account, which are responsible for shifts in transpiration rates throughout the diurnal period. Conversely, the [53] formulation of stomatal resistance is given as the result of a dimensionless function explaining the effects of leaf water potential, vapour pressure deficit, and incident solar flux. In this parameterisation approach, the impact of stomatal resistance is influenced by the difference in water potential between mesophyll and epidermal leaf layers, known as the leaf–atmosphere vapor pressure. The vapor pressure difference is modelled to proportionally reflect its effect on the resistance of the stomata. The outcome of these operations is converted into units of resistance by multiplication with a constant that is the minimum stomatal resistance, which remains constant for each simulation. It is worth mentioning that the selection of the value used as threshold in the epidermal water potential model input parameter, which is required by the model, is a critical component of the stomatal resistance formulation algorithm in this approach. Below this threshold, which is set by the user, the stomatal resistance increases rapidly as leaf size decreases. After reaching the threshold epidermal water potential, transpiration tends to remain relatively constant until the epidermal water potential crosses the threshold once more, approaching higher values. This phenomenon is known as the “transpiration plateau” by [53], where the authors refer to it as the “flattening” of the transpiration curve. Fluxes that are simulated by the model are expressed in Watts per m^2^ (Wm^−2^) leaf area to facilitate linkage to surface energy balance. The scaling of the parameters that have been simulated from the leaf to the canopy is considered in order to establish a correlation with the surface energy balance. The conversion of flux per unit leaf area to flux per unit surface area is achieved by dividing the leaf area index (LAI) by a “shelter factor”, as defined by [54]. The shelter factor considers the variation in transpiration rates among leaves as solar radiation diminishes beneath the upper canopy. For a more comprehensive understanding of the scaling factor concept, the reader may also refer to [36].

## 3. SimSphere Use in the Last 15 Years

This section builds upon the comprehensive review conducted by [50], which explores various aspects in the applications of SimSphere for studying land–atmosphere interactions based on the model’s initial development to date. Subsequently, the discussion herein shifts towards studies conducted since the [50] review paper and until present, addressing more recent assessments and other analyses in the ongoing exploration of SimSphere’s capabilities. As part of this systematic review conducted herein, Table 2 provides a summary of the key studies covered in the present review along with some of their main findings.

### 3.1. Studies Evaluating LSI Parameters Simulated by SimSphere 

Previous research has directly compared model simulations with ground-based observations or other SVAT models. Research over 20 years using SimSphere has been detailed by [50], so research and applications based on the SimSphere SVAT model over the most recent 15 year period are at the focal point of the studies discussed below. 

**Figure 2:**
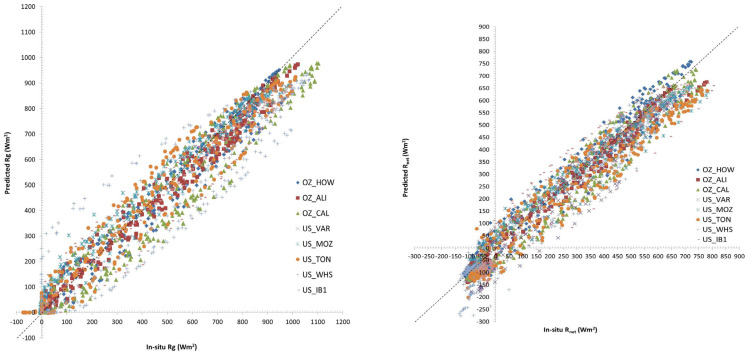
Graphical representation showing the comparisons between SimSphere-predicted and in situ R_g_ and R_net_ fluxes (**left** and **right**, respectively) (adopted from [40]).

Ref. [40] evaluated the model’s ability in predicting key parameters such as LE, H, shortwave incoming solar radiation (R_g_), net radiation (R_net_), air temperature at 50 m (T_air_ 50 m), and air temperature at 1.3 m (T_air_ 1.3 m) for different ecological types located in USA and Australia. The results of the model’s simulations were tested against in situ measurements derived from OzFlux (Australia) and AmeriFlux (USA) networks over 72 chosen days in 2011, representing eight different types of ecosystems. These sites covered a wide range of biome, climatic, and environmental conditions, which allowed contrasting conditions to be included in the model evaluation. Overall, the model simulations showed good to excellent agreement with in situ measurements, especially for LE, H, and T_air_ 1.3 m, followed by T_air_ 50 m with RMSD 39.47, 55.06 Wm^−2^, and 3.23, 3.77 °C, respectively. A systematic underestimation of R_net_ and R_g_ was also found (RMSD 58.69, 67.82 Wm^−2^, MBE −16.46, −19.48 Wm^−2^, respectively). The Nash–Sutcliffe efficiency (NASH) index ranged from 0.72 to 0.99, which indicated a strong model fit to the observed data. Authors reported that woodland ecosystems achieved the highest overall accuracy in simulation, while some ecosystems, such as cropland and grazing pasture, showed poorer simulation accuracy than others. Figure 2 below shows as an example the comparisons between SimSphere-predicted and in situ R_g_ and R_net_ fluxes. 

Later, ref. [55] also evaluated the accuracy of the SimSphere model’s predictions of essential parameters that characterize interactions within the land surface. Specifically, the evaluation focused on the ability of the model to forecast H, LE, R_net_, and T_air_ at 1.3 and 50 m, which was examined over 70 days (10 for each experimental site) at seven CarboEurope network sites corresponding to different biomes and environmental conditions. Overall, the model performed accurate predictions for most of the sites and parameters. According to the statistical metrics, the best results were obtained for H fluxes (average RMSD of 55.36 Wm^−2^ and R^2^ value of 0.83), followed by LE fluxes (average RMSD of 62.75 Wm^−2^) and R_net_ (average RMSD of 64.65 Wm^−2^). The model’s prediction accuracy for T_air_ simulations at 1.3 and 50 m was found to be satisfactory as well (average RMSDs of 4.1 °C and 3.69 °C, respectively). Results indicated that higher vegetation heights appeared to have an effect on simulation accuracy at T_air_ 1.3 m, whereas estimations of T_air_ at 50 m provide better agreement between modelled and measured values. These authors attributed T_air_ temperature closely to vegetation phenology, as it has previously been shown to have a strong influence on the magnitude and extent of air temperature [40]. 

In the same context, ref. [41] examined the SVAT model’s ability to predict R_net_, LE, and H fluxes over 70 days in 2011 at seven CarboEurope network sites, representing various European ecosystems such as forested, grasslands, croplands, and olive plantations. SimSphere performed well in predicting H fluxes, with an average RMSD of 55.36 Wm^−2^. The model provided good agreement for the prediction of LE fluxes and R_net_, with RMSDs of 62.75 Wm^−2^ and 64.65 Wm^−2^, respectively. Findings revealed that among the chosen experiment sites, simulations performed on shrubland land cover consistently displayed low RMSD, particularly for LE and H fluxes. The authors attributed these results to the experimental characteristics, as the sites were located in a water-limited environment, where the effects of transpiration are less pronounced, contributing to higher predictability, especially due to site’s relative homogeneity.

From the above, it becomes evident that several studies aiming at assessing SimSphere’s ability to simulate key variables characterizing LSIs have been performed in recent years. These studies were primarily based on direct comparisons of the model’s predictions with reference data from FLUXNET sites, the largest operational ground measurement network. The model’s performance has so far been assessed for different ecosystem settings worldwide, in Europe, the USA, and Australia. Results have confirmed the models’ ability, demonstrating that the model can be used to facilitate LSIs in both research and practical applications and to support its use in decision making. Based on the accuracies of the model performance, the studies reported overall a good agreement, with acceptable accuracies that vary over different land cover types, which represents a first confirmation of the usefulness of the model as a tool in the study of LSIs. In general, the discrepancies which were reported were attributed, in part, to inherent instrumentation uncertainties and the intricate nature of environmental conditions, which can be attributed to the present architectural design of the model.

### 3.2. Sensitivity Analysis (SA) Studies on SimSphere 

SA becomes particularly crucial for the SimSphere model in order to verify its correspondence with real-world observations and identify the critical input parameters that significantly influence the model’s performance [65]. Hence, as part of model development, it is vital to identify sensitive parameters. This involves comparing simulated outputs with actual observations using statistical methods and sensitivity analysis (SA). Such an analysis plays a key role in validating the model by systematically exploring how changing input parameters affect the model’s output variables [57]. Many studies have been conducted on the SimSphere model aiming to provide an insight into its modelling architecture and coherence. A critical overview of these studies is provided next, along with their key findings.

Ref. [56] pioneered the implementation of SA in the SimSphere model with the application of the Bayesian Analysis of Computer Code Outputs (BACCO) method. BACCO is a sophisticated approach employed in global sensitivity analysis (GSA) within environmental modelling. It is designed to handle multiple sources of uncertainty affecting model performance and provides a comprehensive means of assessing a model’s sensitivity and stability [66]. The method first constructs a statistical emulator of the model using training data points and second uses this emulator to perform SA. In contrast to conventional GSA approaches (e.g., Monte Carlo-based methods), BACCO is characterised by its efficiency, requiring fewer model runs, making it orders of magnitude faster, and allowing the analytical derivation of sensitivity measures directly from the emulators [67]. In addition, BACCO provides a simple way to rank the model inputs according to their importance, based on the computed percentage contribution of the main effect of each input to the total variance of a given parameter predicted by the model. The percentage variance component associated with each input quantifies the amount by which its main effect contributes to the total output variance. In addition, the total effects are computed, which provide insightful information on the degree of input parameters interactions [60]. A detailed description and insights into the BACCO method can be found in [67,68,69].

In their study, ref. [56] performed experiments with SA on SimSphere using a Gaussian process emulator, examining 30 input parameters. The study used LP-tau sampling and produced a design space of 400 simulations of the model. Emulator accuracy was evaluated based on statistical measures and showed an excellent performance. The SA results showed a high sensitivity for all model outputs to a limited number of model inputs, particularly to slope, aspect, surface soil moisture (M_o_), and Fr. Additional influential model inputs included the vegetation height, surface roughness, and substrate climatological mean temperature. Soil parameters were also identified as significant. Although the study provided first insight into the model’s structure and a comprehensive sensitivity mapping of key target variables to inputs, the study assumed normal probability distribution functions (PDFs) for all inputs and outputs, which, as was also noted by the authors, may not always be the case in nature. However, this study marked the first implementation of the BACCO method to a complex simulation SVAT model, namely, SimSphere.

Along the same lines, ref. [57] used BACCO GEM to perform another SA on SimSphere, evaluating the sensitivity of critical model outputs, assuming uniform PDFs for both model’s inputs and outputs. Top of FormSubsequently, the GSA expanded the scope of SA to evaluate new model output parameters that had not been explored in previous relevant SA studies, including the daily average non-evaporative fraction (NEF_daily_), daily average radiometric temperature (Trad_daily_), and daily average evaporative fraction (EF_daily_). The use of uniform probability density functions revealed varying parameter sensitivities for different model outputs, such as Rn_daily_, LE_daily_, H_daily_, Tair_daily_, and Mo_daily_. Findings revealed that aspect, slope, Fr, and M_o_ consistently influence these outputs, explaining significant variances. Additionally, new model parameters (EF_daily_, NEF_daily_, and Trad_daily_) maintain accurate emulator performance. The results indicated that a limited set of input parameters significantly influenced the model’s outputs. Key input parameters which were identified as most sensitive included the slope and aspect, which demonstrated the highest impact on simulating nearly all examined model outputs. Fr and M_o_ were also found to be sensitive for two out of five model outputs each (i.e., for LE_daily_ and H_daily_ and for LE_daily_ and Mo_daily_, respectively) and surface roughness, vegetation height, and soil parameters to a lesser extent. Another important finding of the authors’ study was that the choice of PDFs for input parameters had a minimal effect on the sensitivity of model outputs, confirming previous conclusions on the sensitivity of SimSphere.

In another study conducted by [58], the model’s sensitivity was tested using data acquired from a diverse experimental site and climate regime, providing valuable insights into the model’s coherence. Particularly, experiments in previous studies were limited to a specific atmospheric sounding setting [56,57]. In this study, a different region’s atmospheric sounding was used to perform SA. This allowed for the examination of the sensitivity of key variables in response to variations in the atmospheric sounding profile, considering different input/output PDFs and focusing on specific model outputs (Rn_daily_, LE_daily_, H_daily_, Tair_daily_, Mo_daily_, EF_daily_, NEF_da_ily, and Trad_daily_). In addition, the authors examined the effects of different PDFs for the inputs and outputs of the model, a facet not previously exhaustively investigated. By extending the analysis to a different climate regime, the study validated the global-scale coherence of the model. In accordance with previous studies, this study found that model outputs are notably influenced by only a limited number of inputs. Overall, aspect and slope were reported as the parameters with the greatest influence on the simulation of the model outputs examined in this study. Several model outputs examined were found to be sensitive to vegetation parameters (e.g., Fr, vegetation height), surface roughness, and soil moisture. The study also concluded that employing different PDFs for the model inputs and outputs did not significantly affect the mapping of the most sensitive inputs and interactions. However, it did have an impact only in terms of the absolute numbers used to assess the SA, as computed by the BACCO method.

Later, ref. [59] conducted a GSA on SimSphere, employing a meta-modeling approach based on Bayesian theory by investigating the sensitivity of previously examined parameters [58] parameters with the addition of two parameters examined for the first time, namely, daily average longwave upwelling radiation (L_up_) and daily average longwave downwelling radiation (L_down_). The study initially focused on the effects of uniform PDFs for the inputs of the model and the subsequent SA of key parameters simulated at different output times. Similarly, to previous studies, topographic input parameters, vegetation-related factors, and M_o_ significantly influenced simulated model outputs, even when the model is executed at different times of the day. Particularly, slope and aspect emerge as significant parameters given their influence on R_g_, energy fluxes and near-surface hydrological processes. Vegetative height, surface roughness, and M_o_ were found also to be significant. Moreover, the analysis indicated that the influence of specific parameters on outputs of the model can vary throughout the day. For instance, the importance of topographic parameters such as aspect and slope may change, and the influence of parameters like M_o_ and vegetation height can be time dependent. This temporal variability highlighted the need for the consideration of the time of model simulation in parameterisation and also in setting initial conditions.

**Figure 3:**
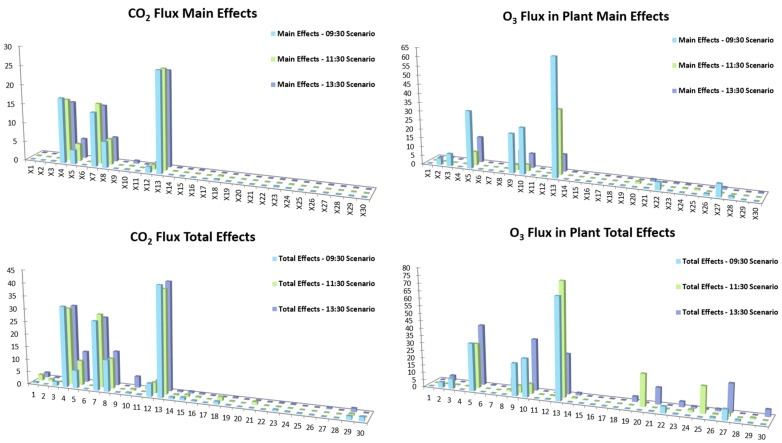
Results of the BACCO GEM SA method are shown according to different model simulations. The vertical axis shows the relative influence of each input parameter on the model output, while the horizontal axis shows the 30 input parameters studied. The input parameters’ contributions to the variance decomposition of the main effects and total effects are presented (**left**), as well as SA results for the CO_2_ flux model output (**right**) and the O_3_ Flux in the plant model output (adopted from [43]).

In the context of taking into account the temporal variability of key parameters, ref. [43] employed the GEM-SA tool to evaluate the sensitivity of various model outputs to changes in input parameters (Figure 3). In particular, authors focused on ambient [CO_2_], the rate of CO_2_ uptake by the plant, the ambient [O_3_], the flux of O_3_ from the air to the plant/soil boundary, and the flux of O_3_ taken up by the plant, considering (3) different time scenarios. Both the cases of normal and of uniform PDFs for the inputs and the outputs of the model have been investigated. Results indicated that only a small subset of model inputs showed a significant influence on model outputs, mainly in vegetation-related factors, CO_2_, and O_3_ fluxes. The external [CO_2_] and its influence on [O_3_] and leaves in the air were also highlighted as significant input parameters. Other important factors were the influence of [O_3_] and [Ca] in the atmosphere, particularly in relation to CO_2_ canopy output. Parameters such as vegetation height, LAI, Fr, and cuticle resistance (CR) were identified as the most sensitive inputs for most model outputs.

Later, ref. [60] used the BACCO method to assess the impact of various input parameters, particularly focusing on the sensitivity of daily average air temperature at 50 m (Tair_daily_ at 50.). The study highlighted aspect, slope, and Fr followed by vegetation height and M_o_ as the most significant input parameters. These results were in line with previous studies that have also identified the same set of sensitive parameters related to T_air_ (aspects, slope, Fr, vegetation height, M_o_, and surface roughness) on T_air_ by assessing the relative contributions of various model inputs. T_air_ was found to be sensitive to the parameters to which incoming solar radiation is sensitive. In particular, the Fr cover is important because it affects the LE and H fluxes, which in turn have an effect on the radiation dynamics and the air temperature. Similarly, ref. [70] also examined the sensitivity of Tair_daily_ at 50 m in the SimSphere SVAT model to various input parameters and identified slope, aspect, and fractional vegetation cover as most significant factors affecting T_air_ daily (50 m). The expected sensitivity of T_air_ to parameters like solar radiation and vegetation cover due to their influence on heat fluxes was also confirmed. 

In conclusion, concerning the SA studies implemented on SimSphere, it is evident that a series of such studies have been recently implemented on it. The common characteristic between all these SA studies was the implementation of a global sensitivity analysis (GSA) using the BACCO method, a sophisticated SA approach in environmental modelling based on the development of an emulator. All SA studies performed confirmed SimSphere structure and architectural coherence, identifying a limited number of model inputs as the most sensitive ones to the simulation of key parameters characterising LSIs by the model. In particular, the most sensitive model inputs in most cases included the slope and aspect along with parameters such as Fr and M_o_, which notably are easily obtainable parameters from EO datasets. The selection of PDFs for the input and output parameters was found to not significantly change the overall SA results, even though it might have an effect on the absolute sensitivity measures. In addition, temporal variability in parameter influences on model outputs was observed, highlighting the importance of considering the time of simulation.

### 3.3. Studies Focusing on Integrating EO Datasets with SVAT Model

The SimSphere model has also been proposed to be used synergistically with remote sensing datasets to provide spatiotemporal estimates of H/LE fluxes as well as M_o_ via the so-called “triangle” method [71]. The primary motivation in incorporating EO data is the inherent challenge of acquiring precise spatiotemporal variability information on these parameters, particularly across large and heterogeneous areas. In this context, the current section reviews the synergistic use of SimSphere with EO data, with a focus on the “triangle” method. 

**Figure 4:**
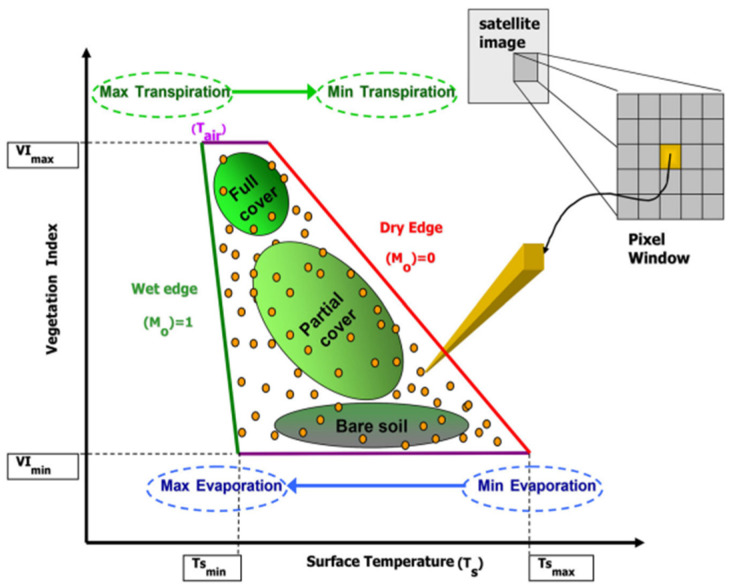
The Ts/VI feature space scatterplot’s key descriptors and physical interpretations are summarized (adopted from [50]).

This method is called the “triangle” method due to the triangular (or trapezoidal) shape that emerges when a satellite-derived vegetation index (VI) is plotted against surface temperature (T_s_) over a rather heterogeneous area (Figure 4). This triangular feature space is a result of the complex interplay between Ts and VI, with vegetation affecting Ts differently in vegetated areas compared to bare soil regions. The terms “dry edge” or “warm edge” within the “triangle” or “trapezoidal” scheme correspond to the highest temperature points, which include different bare soil and vegetation fractions. Similarly, the “wet edge” or “cold edge” depicts water availability relative to vegetation conditions. The “cold edge” and “warm edge” are essentially the boundaries of the triangle which indicate the physical constraints that control these processes. Pixels with the same VI have the greatest evaporative cooling at the lowest Ts, while pixels with the highest Ts have the opposite effect. A thorough conceptual explanation of the method’s foundation can be found in [38,39,72]. The role of changing soil thermal inertia in influencing T_s_ is highlighted by the trapezoidal shape that can appear in the scatterplot, rather than a perfect triangle. The factors that influence the formation of T_s_/VI, such as soil evaporation, leaf stomatal resistance, Fr, surface moisture status, local meteorology, and incoming radiation, are critical for the reliable interpretation of EO data.

Researchers have used the Ts/VI scatterplot to uncover biophysical properties such as surface resistance to moisture fluxes, Mo, and LE/H fluxes. The potential to extend these concepts to regional scales and operational use has been explored, emphasising the scalability of the Ts/VI approach using remote sensing satellites [39,50,61,71,72]. 

For example, ref. [61] explored the integration of advanced along track scanning radiometer (AATSR) and advanced spaceborne thermal emission and reflection radiometer (ASTER) satellites with SimSphere using the “triangle” method to estimate SMC across different ecosystems in Europe. In this study, the SimSphere model, coupled with the triangle method, provided spatial estimates of SMC. Standard statistical metrics were used to evaluate the accuracy against in situ measurements from the CarboEurope flux network as a reference. It was found that AATSR data were more accurate than ASTER data. Even so, the “triangle”-derived SMC using ASTER imagery exhibited an RMSD of 0.19 vol vol^−1^, slightly exceeding the operational accuracy requirement of 0.10 vol vol^−1^. Conversely, a significant improvement was observed when AATSR data were used, resulting in an RMSD of 0.06 vol vol^−1^. Correlation coefficients showed a strong agreement between in situ measurements and both schemes (ASTER R = 0.561, AATSR R = 0.844), with AATSR performing better than ASTER.

Later, ref. [62] used the SimSphere model to simulate the complex interactions within the land surface temperature/fractional vegetation coverage (LST/FVC) space. In their study, a two-step trapezoid instead of a “triangle” method was proposed, where vegetated surface temperature (T_v_) varies after Ts due to the ability of vegetation to absorb deep soil moisture to maintain transpiration. The ability of the two-stage trapezoid to match the simulated LST/FVC space and estimate EF using the HiWATER dataset and MODIS products was compared to that of the traditional trapezoidal LST/FVC space. The SimSphere model conducted simulations varying FVC, SSM availability, and root zone soil moisture availability (RSM) from 0 to 1, aiming to compute the integrated LST of corn vegetation and silty loam soil mixtures at a grid-cell scale and create a simulated LST/FVC space. According to the results of this study, the two-stage trapezoid more closely corresponded with the simulated LST/FVC space. Notably, this study leveraged the capabilities of the SimSphere model to perform a comprehensive validation analysis.

Recently, ref. [63] evaluated the triangle method using AATSR data at 1 km mesoscale resolution in order to determine how atmospheric correction and heterogeneity on the land surface affect the accuracy of the predictions. Particularly, the study explored the use of EO AATSR data coupled with SimSphere within the “triangle” method to obtain H, LE, and SMC and also the daytime average LE and H fluxes (as expressed from the ratios of LE/Rn and H/Rn, respectively) in different ecosystems in Europe. The impact of atmospheric correction on the input datasets was examined using both non-atmospheric (1P) and atmospherically corrected (2P) AATSR data spanning from 2007 to 2011 (a total of 47 imageries) across 12 CarboEurope monitoring network sites. For all simulated parameters, the results showed that the prediction accuracy was significantly improved with data at level 2P. In particular, higher prediction accuracy was obtained for LE (R = 0.72 versus R = 0.91), SMC (R = 0.76 versus R = 0.84), and H (R = 0.30 versus R = 0.68) fluxes, followed by H/Rn (R = 0.0.57 versus 0.63) and LE/R_n_ (R = 0.49 versus 0.63). The average improvement in RMSD across these parameters for 2P- compared to 1P-level products was reported to be ~15 Wm^−2^. The results presented in this study indicated that there has been a notable improvement in prediction accuracy with the 2P-level product. Still, the authors acknowledged challenges in their study implementation, including geolocation errors and surface heterogeneity, which may introduce potential sources of error. Overall, the importance of atmospheric correction in improving the accuracy of predictions was highlighted.

More recently, ref. [64] used SimSphere to assess the impact of a recently proposed Priestly–Taylor equation, namely Sun2021 compared to the previously suggested Sun2016 method, to determine the wet edge boundary of the LST/FVC space for the prediction of soil moisture and air temperature. The study used SimSphere model-simulated data, satellite data from MODIS, air temperature from NLDAS, and SM observations from aircraft campaigns (SMAPVEX16-IA, SMAPVEX16-MB, and SMAPVEX12). Specifically, SimSphere was used to simulate key parameters, allowing for a systematic comparison between the proposed Sun2021 and the traditional Sun2016 approaches. The findings suggested that the new PT equation outperformed the traditional one in defining LST/FVC space boundaries. The results also demonstrated the ability of SimSphere to be used for intercomparison between models.

All in all, from the above studies, it becomes evident that the SimSphere model has also demonstrated its robustness so far and in its integration of EO data, in particular in the context of the “triangle” method. The incorporation of satellite-based data enhances the model’s capabilities, making it a valuable tool for understanding land–atmosphere interactions and providing critical information for various environmental applications. The model has proven to be effective in providing accurate spatiotemporal variability information for SMC and energy fluxes and water cycle-related fluxes over diverse landscapes. The Ts/VI scatterplot’s triangular feature space offers knowledge on the intricate relationships between surface temperature and vegetation. An important aspect of the model is SimSphere’s adaptability, demonstrated by its ability to be applied on a variety of spatial scales and to adapt to different ecosystems. Another important aspect is the model’s contribution to model intercomparison efforts. 

## 4. Recent Developments in SimSphere’s Modelling Architecture

**Figure 5:**
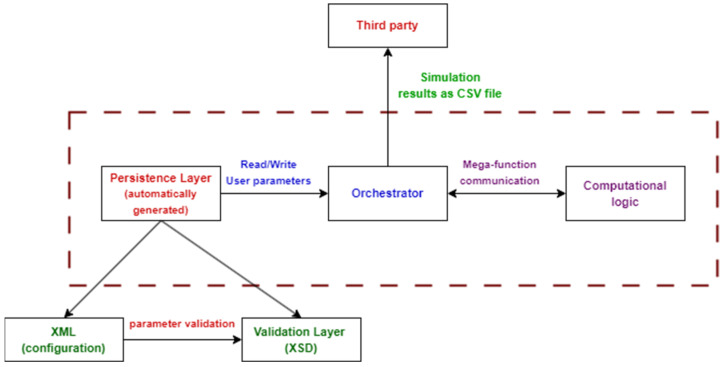
The new user-friendly architecture of SimSphere–SOA.

Recent developments in the SimSphere SVAT model’s architectural component have so far focused on developing the model based on service-oriented architecture (SOA) principles in order to improve model’s usability and applicability [37]. The computational logic layer contains an orchestrator that reads XML via a mega-function interface and exports computation results to CSV for third-party applications (Figure 5). The architecture supports two CSV endpoints: one for time-based simulations and another for scenario exploration. The architecture is designed to be stateless, making it appropriate for high-performance computing (HPC) environments and allowing for scalability via stateless clusters. SimSphere–SOA also improves the ease of use of the SA tool, which helps in understanding the model’s behaviour and identifying critical input parameters. In addition, SimSphere–SOA addresses challenges in the original toolkit, such as the lack of model inversion data export, the complexity of manual configuration, and issues related to third-party interfaces and high-performance computing. SimSphere–SOA is built on the principles of SOA to enable adaptable simulations. The serialisation layer generated from the 1.0 document facilitates the integration of XML data into appropriate classes. It is worth noting that this approach is four times smaller in size than manually generated Java code and does not require any prior knowledge of Java. These updates include the integration of new functionalities, resulting in a significant enhancement of the model’s capabilities.

These new functionalities enhance the model’s architectural component to accommodate heterogeneity within its simulations and incorporate additional physical processes, such as run-off and percolation, using sophisticated EO satellite data and novel image analysis methods. 

## 5. Discussion

Concerning the key results of the present review, first and foremost, it is clear that a significant number of studies have been performed using the model in the last 15 years covering a wide range of application areas. In particular, the successful application of SimSphere to various types of ecosystems has, to some degree, confirmed its ability to respond to the description of the complex physical processes of the natural environment, producing results that are in alignment with other SVATs [42,64]. In addition, the extensive number of SA studies implemented on SimSphere have clearly enhanced our understanding of the model’s sensitive parameters and structural and architectural coherence. A very important finding in that respect is the fact that most SA studies identified the influential model inputs as the ones which are easily accessible through EO-based datasets or products. Furthermore, SimSphere’s application with EO data, especially using the “triangle” approach, provides valuable insights into the way SimSphre can be used towards deriving spatiotemporal estimates of key parameters characterizing LSIs, namely SMC, LE, and H fluxes. Last, but not least, the SimSphere model has undergone significant improvements in recent years, broadening its functionality and applicability in both teaching and research.

The SimSphere SVAT model carries out all the advantages of SVAT models. As such, it is able to comprehensively analyse a large array of parameters characterising LSIs associated with the hydrological, radiative, and physical domains of the Earth’s system dynamics. Furthermore, SimSphere represents an effective method for examining interactions and physical processes and executes different scenarios when studying LSIs at exceptional temporal resolution, which is consistent with atmospheric and dynamical physical processes. In addition, a unique characteristic of SimSphere, to some extent, is the fact that it can also be integrated with RS data via the so-called “triangle” method and can be executed in an HPC environment. 

Yet, SimSphere is also characterised by some limitations, many of which are inherent to almost all SVAT models. In particular, a certain limitation is associated with the large amount of input parameters required in its parameterisation and the actual geographical scale represented in the parameterisation, which may hinder the model’s implementation under certain circumstances (e.g., highly fragmented ecosystems). Also, given the computational complexity to perform data processing operations and simulations, their application would require some technical expertise by the user.

There is still potential for improvements in different aspects of the model; thus, future research will focus towards this direction. More specifically, the further development of SimSphere’s use in ecological settings with varying physical and climate characteristics will provide a critical contribution towards advancing our understanding of land surface interactions in different environments. The evaluation of the model’s performance in the direction of conducting a detailed assessment of its ability to study LSIs over a variety of vegetation types and environmental conditions using reference data that come from ground observational networks (such as FLUXNET) will provide valuable insights about the model’s coherence and architecture. On this basis, comparing the SimSphere outputs with outputs from other land surface process models is a further priority research topic. Another important aspect would be the expansion of the model’s functional platform by including physical processes such as runoff and/or percolation. The automation of aspects linked to the synergistic use of the SimSphere model with the EO datasets via the “triangle” method can represent another important direction of future research linked to the model. Last, but not least, the linkage of the model platform with open-source image processing platforms would offer versatility in its application and compatibility with various EO data methods.

## 6. Concluding Remarks

The aim of the present communication was two-fold: first, to provide a critical evaluation of the recent use of the SimSphere SVAT model within the scientific and wider user community; second, to provide information on recent software developments implemented to SimSphere. This study extends the literature review provided by [50] based on SimSphere applications, where early applications of the SimSphere model since its initial development were extensively covered. As such, the present review shifted the focus to more recent studies over a 15 year period, providing insights into ongoing evaluations and analyses exploring the evolving process of SimSphere. 

All in all, the model’s multidisciplinary nature further underscores its adaptability and promising potential as a toolkit either as a stand-alone tool or coupled with novel EO-based datasets to study LSIs in a multi-faceted way. Yet, there are challenges that still remain to be addressed concerning the future model use and its further dissemination, as already noted. Future work within our group is directed towards some of those directions and also aim to contribute towards extending the model’s use by the wider community.

## Figures and Tables

**Table 1:** The model architecture is represented by an electrical analogy based on Ohm’s law using the coefficients and variables taken from SimSphere, as shown in Figure 1b.

Name	Variable	Units
Air vapour pressure in the atmosphere	e_a_	Mbar
Leaf–air boundary vapour pressure	e_af_	Mbar
The saturation vapour pressure at the temperature of the leaf	e_L_ (T_L_)	Mbar
Flow of water from soil to leaf	F_w_	Wm^−2^
Foliage sensible heat flux	H_f_	Wm^−2^
Soil sensible heat flux	H_g_	Wm^−2^
Foliage latent heat flux	L_e_E_f_	Wm^−2^
Soil latent heat flux	L_e_E_g_	Wm^−2^
Air resistance in surface layer	r_a_	sm^−1^
Resistance of heat and water vapour flux for interleaf air spaces	r_af_	sm^−1^
Air resistance between the ground and the interleaf air spaces	r_ag_	sm^−1^
Air resistance in transition surface layer	r_b_	sm^−1^
Soil resistance from the substrate	r_g_	sm^−1^
Leaf resistance	r_L_	sm^−1^
Soil water content of the root zone	θ_v_	cm^3^ cm^−3^
Surface soil water content	θ_vo_	cm^3^ cm^−3^
Soil water potential	ψ_g_	Bar
Mesophyllic leaf water potential	ψ_L_	Bar
Air temperature of the surface layer	T_a_	Kelvin
Temperature of the interfoliage air spaces	T_af_	Kelvin
Temperature of the ground surface	T_g_	Kelvin
Temperature of the leaf surface	T_L_	Kelvin
Root resistance	Z_root_	Bar (Wm^−2^)^−1^
Stem resistance	Z_stem_	Bar (Wm^−2^)^−1^
Soil root surface resistance	Z_g_	Bar (Wm^−2^)^−1^
Shielding factor	σ_f_	Unitless

**Table 2:** Summary of main studies performed using the SimSphere SVAT model.

Study Group	Study Details	Study Objective	Key Findings	
**Studies evaluating the SVAT model outputs**	[40]	Evaluation of R_g_, R_net_, LE, H, T_air_ 1.3 m, and T_air_ 50 m using in situ measurements from eight sites within the AmeriFlux (USA) and OzFlux (Australia) monitoring networks.	Varied simulation accuracy across land cover types. High agreement for LE (RMSD 39.47 Wm^−2^), H (55.06 Wm^−2^), T_air_ 1.3 m (3.23 °C), and T_air_ 50 m (3.77 °C). Slightly underestimated R_net_ (RMSD 58.69 Wm^−2^, MBE −16.46 Wm^−2^) and R_g_ (RMSD 67.82, MBE −19.48 Wm^−2^).	
	[55]	Evaluate R_net_, LE, H, T_air_ 1.3 m, and T_air_ 50 m against in situ measurements acquired from seven CarboEurope (European) network sites.	High agreement observed for H fluxes (RMSD 55.36 Wm^−2^, R^2^ 0.83), LE fluxes (62.75 Wm^−2^), and R_net_ (64.65 Wm^−2^). Increased vegetation reduced accuracy at T_air_ 1.3 (RMSD 4.1 °C) but enhanced model agreement at T_air_ 50 (RMSD 3.69 °C).	
	[41]	Evaluate R_net_, LE, and H against in situ data acquired from seven CarboEurope (European) network sites.	Shrubland consistently showed low RMSDs in all outputs. Highest agreement for H (55.36 Wm^−2^), followed by LE (62.75 Wm^−2^) and R_net_ (64.65 Wm^−2^).	
**Studies based on SA of the model**	[56]	GSA based on BACCO method for Rn_daily_, LE_daily_, H_daily_, Tair_daily_ and Mo_daily_.	Initial BACCO GSA implementation in SimSphere. Highly sensitive model inputs: aspect, Fr, M_o_, and slope; lower sensitivity to roughness, vegetation height, and substrate mean temperature.	
	[57]	EF_daily_, NEF_daily_, and Trad_daily_ Assumption of normal PDFs for the inputs/outputs.	Most sensitive inputs: aspect, slope, Fr, and M_o._ PDFs had minimal impact on model output sensitivity.	
	[58]	Rn_daily_, LE_daily_, H_daily_, Tair_daily_, Mo_daily_, EF_daily_, NEF_daily_, and Trad_daily._ Assumption of uniform PDFs for the inputs/outputs.	Most sensitive inputs: slope, aspect, height, Fr, and roughness. Aspect and slope most influential across all model outputs.	
	[59]	Rn_daily_, Rg_daily_, LE_daily_, H_daily_, Tair_daily_, Mo_daily_, EF_daily_, NEF_daily_, Trad_daily_ L_down_, and L_up._ Assumption of uniform PDFs for the inputs/outputs.	Most sensitive inputs: slope and elevation, followed by aspect, Fr, vegetation height, and M_o_. PDFs had minimal impact on model output sensitivity. Temporal variability observed in parameter influence on model outputs.	
	[43]	Evaluation of [CO_2_], CO_2_ fluxes, [O_3_], and O_3_ fluxes. Assumption of normal and uniform PDFs for the inputs/outputs.	Most sensitive inputs: LAI, Fr, CR, and vegetation height. Limited model inputs significantly influence outputs, mainly related to vegetation, CO_2_, and O_3_ fluxes.	
	[60]	Sensitivity of the Tair_daily_ at 50 m. Assumption of uniform PDFs for the inputs/outputs.	Most sensitive inputs: slope, aspect, and Fr. Lesser contributors: M_o_ and vegetation height.	
**Studies focusing on the coupling EO datasets with the SVAT model**	[61]	Estimation of SMC via the triangle method using data from AATSR/ASTER. Measurements obtained from seven CarboEurope (European) network sites.	Superior performance of “triangle”-derived SMC with AATSR over ASTER.	
			ASTER: RMSD = 0.19 vol vol^−1^ MBE = 0.08 vol vol^−1^ R = 0.56	AATSR: RMSD = 0.06 vol vol^−1^ MBE = 0.01 R = 0.84
	[62]	Validation of the two-stage trapezoid.	Two-stage trapezoid model aligns closely with simulated LST/FVC space, validated comprehensively using SimSphere.	
	[63]	Estimation of LE, H, SMC via the triangle method using EO AATSR 1P and 2P data and assessed atmospheric correction effects on accuracy.	SimSphere with EO AATSR data used for process simulation. “Triangle” method applied, comparing 1P and 2P AATSR data. 2P level product improved flux prediction accuracy for all parameters (SMC, LE, H H/R_n_, and LE/R_n_).	
	[64]	An intercomparison analysis of models, based on SimSphere’s simulations.	SimSphere model to evaluate Sun2021 for LST/FVC space. Results underscore SimSphere’s role in model intercomparison.	
